# Inoculation with Ericoid Mycorrhizal Associations Alleviates Drought Stress in Lowland and Upland Velvetleaf Blueberry (*Vaccinium myrtilloides*) Seedlings

**DOI:** 10.3390/plants10122786

**Published:** 2021-12-16

**Authors:** Deyu Mu, Ning Du, Janusz J. Zwiazek

**Affiliations:** 1 Center of Landscape Architecture, Shandong Jianzhu University, Jinan 250000, China; mudeyu@yeah.net; 2 Institute of Ecology and Biodiversity, School of Life Sciences, Shandong University, Qingdao 266237, China; ndu@sdu.edu.cn; 3 Department of Renewable Resources, University of Alberta, Edmonton, AB T6G 2E3, Canada

**Keywords:** ericoid mycorrhizae, drought resistance, growth, gas exchange, water potentials

## Abstract

Although velvetleaf blueberry (*Vaccinium myrtilloides*) is usually associated with sandy (upland) areas of the North American boreal forest, lowland populations can be also found in bogs, suggesting possible adaptations to different site conditions. In this study, we examined the role of ericoid mycorrhizal (ERM) fungi in conferring drought resistance to the upland and lowland velvetleaf blueberry seedlings. The seedlings were inoculated with four ERM fungi (*Pezicula ericae*, *Pezoloma ericae*, *Meliniomyces variabilis*, and *Oidiodendron maius*) isolated from the roots of ericaceous plants and grown under controlled environmental conditions in sterilized soil. The inoculated and non-inoculated (inoculation control) plants were subsequently subjected to three cycles of drought stress by withdrawing watering. Lowland plants appeared to benefit relatively more from mycorrhizal colonization, compared with the upland plants, in terms of plant growth and drought survival. After three weeks of treatments, the dry weights of non-inoculated well-watered upland plants were higher compared to the non-inoculated lowland plants. However, these differences were offset by the inoculation of plants with ERM fungi, some of which also significantly improved drought resistance characteristics of the upland and lowland plants. There were no major differences in the effects of different ERM fungal species on drought responses of upland and lowland plants. Of the examined ericoid mycorrhizal fungi, inoculation with *Pezicula ericae* was the most effective in conferring drought resistance characteristics to both upland and lowland seedlings and helped maintain higher shoot water potentials, net photosynthetic, and transpiration rates in plants subjected to drought stress.

## 1. Introduction

Ericaceae is a large family of small plants and shrubs commonly growing in acidic infertile soils [1]. Ericaceous plants form symbiotic associations with ericoid mycorrhizal (ERM) fungi. These associations are characterized by the formation of intracellular hyphal coils in the root epidermis and short hyphal extensions from the root surface [2]. Although the taxonomic status of ERM fungi has received considerable attention, compared with the ectomycorrhizal and arbuscular mycorrhizal associations, far less is known about the functional significance of various ericoid fungi within the plant roots and about the responses of ERM associations to various environmental conditions [3,4]. An application of molecular techniques has revealed a high diversity of ERM fungi that may colonize roots of a single ericaceous plant [5,6]; however, the contributions of different ERM fungi to plant responses to environmental factors remain obscure.

Physiological studies of the symbiosis between ERM fungi and their ericaceous hosts are limited and have been largely conducted with *Rhizoscyphus ericae* (formerly *Hymenoscyphus ericae*). There is especially little information concerning the effectiveness of ERM fungi in conferring stress resistance to ericaceous plants [4]. A recent study reported that *Oidiodendron maius* and *Meliniomyces variabilis* improved salt tolerance of the three examined ericaceous species of plants and their effects on the different growth, and physiological parameters varied depending on the fungal and plant species [7]. However, little is known about the effectiveness of ERM associations on the responses of ericaceous plants to soil moisture conditions. Since both ectomycorrhizal (ECM) and arbuscular mycorrhizal (AM) associations play important roles in controlling plant water relations [8,9], it is plausible that ERM fungi are involved in the adaptation of ericaceous plants to different soil moisture conditions.

The alleviation of adverse soil factors by AM [9,10] and ECM [8] associations has often been explained by an enhancement of root water uptake and transport [11,12,13,14]. This effect of mycorrhizal associations is partly due to the enhancement of root [13,14] and fungal [14] aquaporin activity and expression, and the resulting increases in cell and root hydraulic conductivities [15]. However, these effects vary between the plant and mycorrhizal fungal species [16,17] and are not always consistent [18]. Currently, there have been no reports demonstrating the effectiveness of ERM associations in enhancing drought resistance of ericaceous plants.

Growth habitats of ericaceous plants range from dry to wet. Velvetleaf blueberry (*Vaccinium myrtilloides*) is usually associated with dry sandy areas of the North American boreal forest; however, distinct populations can be also found in wet hummocky areas in bogs [19]. In this study, we examined the effects of ERM fungi on the responses of upland and lowland velvetleaf blueberry to different soil moisture conditions to ascertain whether mycorrhizal associations can contribute to the successful colonization of dry sites by the upland blueberries.

In the present study, we isolated ERM fungi from the roots of ericaceous plants growing in the boreal forest sites where the seeds of velvetleaf blueberry plants were collected for the experiment. We subsequently inoculated with these fungi the roots of velvetleaf blueberry grown from the seeds of plants that had been harvested from a dry sandy soil forested area (upland blueberry) and from the hummocky bog (lowland blueberry) to examine the responses of plants to drought stress. The main objectives of the study were to determine whether (i) plants grown from seeds collected from the upland and lowland velvetleaf blueberry vary in their responses to drought stress, (ii) ERM associations enhance drought resistance of plants, and (iii) ERM fungi differently affect the responses of upland and lowland seedlings to drought. We hypothesized that ERM associations would play a significant role in conferring drought protection to plants, but their effectiveness will vary between the upland and lowland seedlings depending on the ERM fungal species.

## 2. Results

### 2.1. Volumetric Soil Water Content

**Figure 1:**
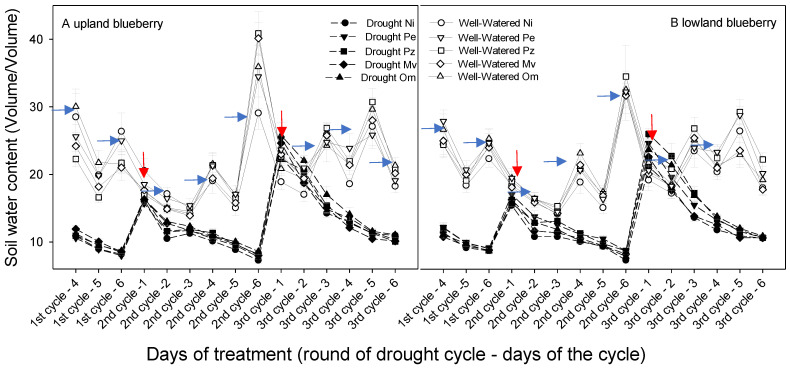
Volumetric soil water content in pots with upland (**A**) and lowland (**B**) velvetleaf blueberry plants that were either non-inoculated (mycorrhizal control) or inoculated with different ERM fungi (Pe-*Pezicula ericae*, Pz-*Pezoloma ericae*, Mv-*Meliniomyces variabilis*, Om-*Oidiodendron maius*), and Ni-non-inoculated. Plants were either well-watered or subjected to three cycles of drought stress. In each cycle, watering was withheld for 7 days and was followed by rewatering to runoff (red arrows). In well-watered treatment, water was added every two days (blue arrows). Means (*n* = 9) ± SE are shown.

In well-watered pots, volumetric soil water content followed similar patterns in all groups of plants and fluctuated between approximately 15 and 40% (Figure 1A,B). Following cessation of watering, the volumetric soil water content progressively declined in pots to below 10% at the end of each cycle in all groups of plants (Figure 1A,B).

### 2.2. Root Colonization

Hyphal coils were observed in the root epidermis of velvetleaf blueberry plants. The colonization intensity (M) in the four ERM inoculation treatments ranged from 50% to 80%, depending on the fungal species and watering treatment (Table 1). In the non-inoculated control plants, M varied from 14.7 to 20.7% (Table 1). The M values in plants inoculated with ERM were significantly higher compared with the non-inoculated control plants (Table 1). Plants inoculated with *Oidiodendron maius* had the highest M, ranging from about 66 to above 78% (Table 1). Well-watered plants had significantly higher M compared with drought-stressed plants as determined by the three-way ANOVA, and there were no significant differences between upland and lowland plants (Table S1).

### 2.3. Plant Mortality

There was no plant mortality in the well-watered inoculated and non-inoculated groups. Approximately 33% of the non-inoculated lowland velvetleaf blueberry and 22% of the non-inoculated upland velvetleaf blueberry seedlings did not survive the three cycles of drought stress treatment (Table 2). Plants inoculated with the ERM fungi had lower mortality rate compared with non-inoculated plants. In the lowland velvetleaf blueberry population, plants inoculated with *Pezicula ericae* and *Pezoloma ericae* had the lowest mortality rates (5.6%), and in the drought-stressed upland velvetleaf blueberry, seedlings inoculated with *Meliniomyces variabilis* had the lowest mortality rate (5.6%).

### 2.4. Dry Weights

**Figure 2:**
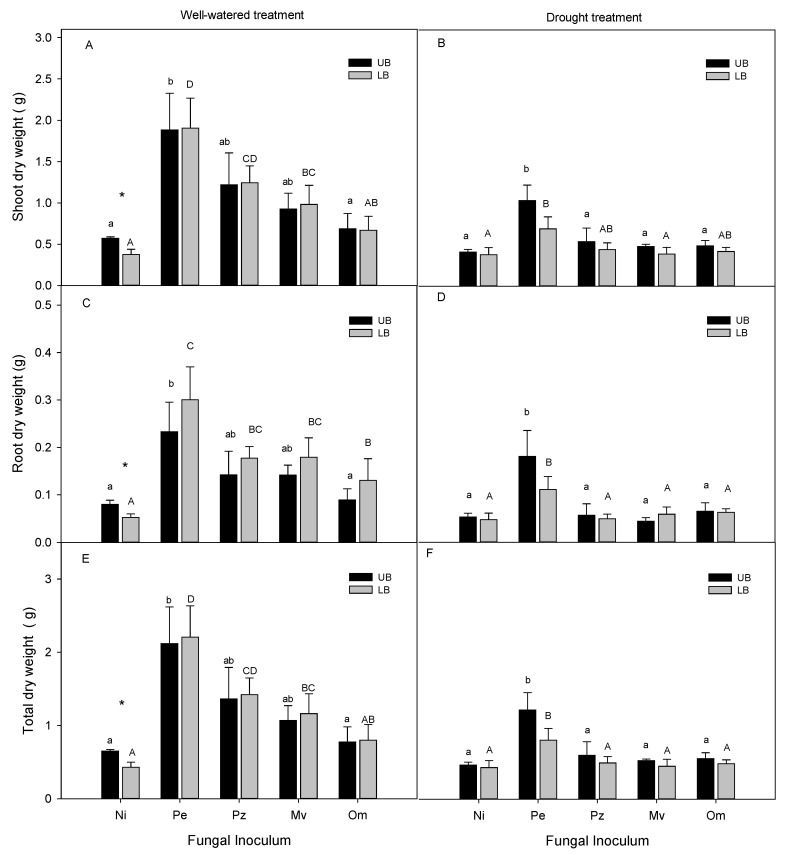
Shoot, root, and total dry weights of upland (UB) and lowland (LB) velvetleaf blueberry seedlings that were either non-inoculated (mycorrhizal control) or inoculated with different ERM fungi. The plants were well-watered (**A**,**C**,**E**) or subjected to drought stress (**B**,**D**,**F**). Bars are means (*n* = 6) ± SE. Different lowercase letters in each subfigure indicate significant differences between the upland blueberry plants subjected to different ERM inoculation treatments, and the uppercase letters in each subfigure indicate significant differences between the lowland blueberry plants subjected to different ERM inoculation treatments (Pe-*Pezicula ericae*, Pz-*Pezoloma ericae*, Mv-*Meliniomyces variabilis*, Om-*Oidiodendron maius*, and Ni-non-inoculated). Asterisks indicate significant difference between UB and LB plants. One-way ANOVA was performed followed by Duncan’s test (*p* ≤ 0.05).

Shoot dry weights of the upland and lowland velvetleaf blueberry seedlings inoculated with the ERM fungi were greater compared with non-inoculated control in both drought and well-watered treatments (Figure 2A,B). Well-watered lowland plants inoculated with *Pezicula ericae*, *Pezoloma ericae* and *Meliniomyces variabilis* had significantly higher shoot dry weights compared with non-inoculated seedlings, while in the upland plants only *Pezicula ericae* inoculation resulted in a significant increase in the shoot dry weights (Figure 2A). Only *Pezicula ericae* significantly enhanced the shoot dry weights in drought-stressed seedlings compared with the non-inoculated treatment (Figure 2B).

In well-watered non-inoculated plants, root dry weights were significantly higher in the upland compared with lowland velvetleaf blueberry (Figure 2C), but there was no difference after the seedlings were subjected to drought stress (Figure 2D). Root dry weights were greater in lowland well-watered plants inoculated with the ERM fungi compared with the non-inoculated plants (Figure 2C). Only *Pezicula ericae* significantly increased root dry weights in well-watered upland plants and in drought stressed upland and lowland plants (Figure 2C).

Similarly to the shoot and root dry weights, the total dry weights of non-inoculated well-watered upland velvetleaf blueberry seedlings were higher compared with the well-watered non-inoculated lowland plants (Figure 2E). Only *Pezicula ericae* significantly increased the total dry weights in well-watered and drought-stressed upland seedlings (Figure 2E,F). In lowland plants, the total dry weights of well-watered seedlings significantly increased compared with non-inoculated plants as a result of inoculation with *Pezicula ericae*, *Pezoloma ericae*, or *Meliniomyces variabilis* (Figure 2E), while in the drought-stressed seedlings only *Pezicula ericae* increased the plant total dry weights (Figure 2F).

### 2.5. Leaf Chlorophyll Concentrations and Gas Exchange

**Figure 3:**
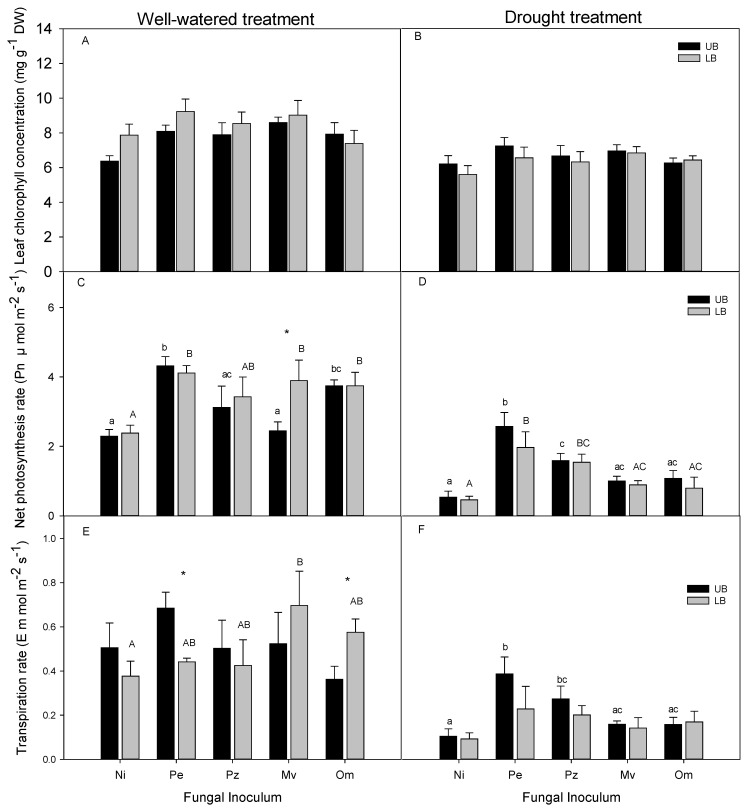
Leaf chlorophyll concentration (**A**,**B**), net photosynthesis (**C**,**D**), and transpiration rate (**E**,**F**) in upland (UB) and lowland (LB) velvetleaf blueberry seedlings inoculated with ERM fungi and subjected to drought stress or well-watered. Data are means (*n* = 6) ± SE. Different lowercase letters in each subfigure indicate significant differences between the upland blueberry plants subjected to different ERM inoculation treatments, and the uppercase letters in each subfigure indicate significant differences between the lowland blueberry plants subjected to different ERM inoculation treatments (Pe-*Pezicula ericae*, Pz-*Pezoloma ericae*, Mv-*Meliniomyces variabilis*, Om-*Oidiodendron maius*, and Ni-non-inoculated control). The plants were subjected to drought stress or well-watered. Asterisks indicate significant difference between the upland and lowland plants. One-way ANOVA was performed followed by Duncan’s test (*p* ≤ 0.05).

There were no significant differences in the leaf chlorophyll concentrations between inoculated and non-inoculated seedlings for the well-watered and drought treatments (Figure 3A,B).

The net photosynthetic rates (Pn) of well-watered upland velvetleaf blueberry seedlings inoculated with *Pezicula ericae* and *Oidiodendron maius* were significantly higher compared with non-inoculated plants (Figure 3C). In well-watered lowland velvetleaf blueberry, seedlings inoculated with *Pezicula ericae*, *Meliniomyces variabilis*, and *Oidiodendron maius* also had significantly higher Pn compared with the non-inoculated control (Figure 3C). After three weeks of the drought treatment, the Pn of drought-stressed velvetleaf blueberry seedlings inoculated with *Pezicula ericae* and *Pezoloma ericae* was several-fold higher compared with the non-inoculated plants in both upland and lowland plants (Figure 3D). Although higher Pn than in non-inoculated plants was also observed in upland and lowland drought-stressed seedlings inoculated with *Meliniomyces variabilis* and *Oidiodendron maius*, the differences were not statistically significant (Figure 3D).

In well-watered treatment, lowland velvetleaf blueberry seedlings inoculated with *Meliniomyces variabilis* had significantly higher E than the non-inoculated plants after 21 days of treatment (Figure 3E). There was no significant difference in E between well-watered inoculated and non-inoculated upland plants (Figure 3E). *Pezicula ericae* had a significantly greater effect on E in well-watered upland, compared with lowland, seedlings while the opposite was observed in seedlings inoculated with *Oidiodendron maius*. Transpiration rates declined as a result of drought stress treatment (Figure 3F). However, there was no significant effect of fungal inoculation on E in drought-stressed lowland plants. The upland seedlings inoculated with *Pezicula ericae* and *Pezoloma ericae* maintained significantly higher E compared with the non-inoculated seedlings when subjected to drought stress (Figure 3F). There were no significant differences in E between the upland and lowland plants for any of the inoculation treatments (Figure 3F).

### 2.6. Shoot Water Potentials

**Figure 4:**
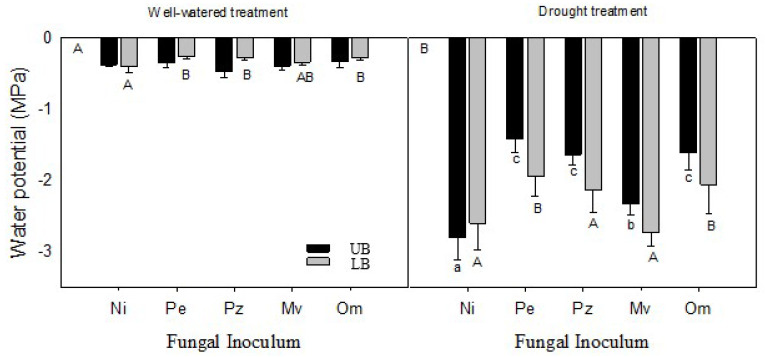
Shoot water potentials in well-watered (**A**) and drought-stressed (**B**) upland and lowland velvetleaf blueberry plants that were non-inoculated (mycorrhizal control) or inoculated with different ERM fungi. Data are means (*n* = 6) ± SE. One-way ANOVA was performed followed by Duncan’s test (*p* ≤ 0.05). Different lowercase and uppercase letters indicate significant differences between inoculation treatments for the lowland and upland plants, respectively. Pe-*Pezicula ericae*, Pz-*Pezoloma ericae*, Mv-*Meliniomyces variabilis*, Om-*Oidiodendron maius*, and Ni-non-inoculated.

Shoot water potentials were similar in the inoculated and non-inoculated well-watered upland plants (Figure 4A). In the lowland velvetleaf blueberry, shoot water potentials of seedlings inoculated with *Pezicula ericae*, *Pezoloma ericae*, and *Oidiodendron maius* were significantly higher compared with the non-inoculated control (Figure 4A).

When exposed to drought stress, shoot water potentials were significantly higher in the upland velvetleaf blueberry inoculated with the four ERM fungi compared to the non-inoculated plants (Figure 4B). In drought-stressed lowland velvetleaf blueberry, shoot water potentials were significantly higher in seedlings inoculated with *Pezicula ericae* and *Oidiodendron maius* compared with the non-inoculated control (Figure 4B).

## 3. Discussion

Although velvetleaf blueberry is commonly found in relatively dry sandy soils, distinct plant populations can also be found in the moister, lowland areas [19]. In the present study, we examined the effects of ERM fungi, which we isolated from the roots of ericaceous plants, on drought resistance of velvetleaf blueberry plants that were grown from the upland and lowland seed sources. The ERM fungi that we used for plant inoculation are distributed world-wide and have been reported to be commonly associated with *Vaccinium* species [8,20,21,22,23,24,25,26].

In our study, root colonization intensity in plants inoculated with the ERM fungi ranged from 50 to 80% depending on the fungal inoculum. Some ERM structures (17% colonization intensity) were also present in the roots of non-inoculated plants and were tentatively identified as *Leohumicola verrucosa*. This heat-resistant fungus [27,28] survives soil autoclaving [27] and is commonly present in a variety of soils [28], including the peat that was used in our study as a growth medium [27].

Our study clearly demonstrated that the ERM colonization had a profound impact on the growth of well-watered plants, especially in the lowland population. Under well-watered conditions, non-inoculated upland plants had higher shoot, root, and total dry weights compared with the lowland plants, but these differences were no longer present in plants inoculated with the ERM fungi. There were also minor or no differences in the examined growth parameters between the upland and lowland plants subjected to drought stress, regardless of the inoculation treatment. Population adaptations to soil moisture conditions have been reported for various plant species [29,30]. Upland populations of rice (*Oryza sativa*) and switchgrass (*Panicum virgatum*) were reported to be more drought resistant than the lowland populations, which was attributed to both drought avoidance and drought tolerance mechanisms [29,31]. However, the role of ERM associations in plant adaptations to environmental conditions has been rarely considered. Since root colonization is affected by soil conditions [32,33], we examined the possibility that drought resistance of lowland and upland plants may vary when colonized by the ERM fungi. However, although *Pezicula ericae* appeared to provide a consistent trend in conferring greater enhancement of drought resistance characteristics to the upland, compared with lowland plants, in terms of growth, gas exchange and shoot water potentials, these differences were not statistically significant.

Mycorrhizal associations commonly increase growth rates of the host plants, also under drought stress conditions [34]. In our study, the effects of fungal inoculates on plant dry weights varied between the fungal species. *Pezicula ericae* was the most effective ERM fungus that significantly increased the plant shoot, root, and total dry weights in well-watered upland plants, while the dry weights of well-water lowland plants were increased by inoculation with *Pezicula ericae*, *Pezoloma ericae*, and *Meliniomyces variabilis*. However, the dry weights of drought-stressed upland and lowland plants were significantly higher compared with non-inoculated control only in upland and lowland plants inoculated with *Pezicula ericae*. *Pezicula ericae* also had a greater impact compared with the other fungal inocula on the examined physiological parameters in both upland and lowland plants under well-watered and drought conditions. Since *Pezicula ericae* was isolated from the upland and *Pezoloma ericae* from the lowland velvetleaf blueberry plants (Table S2), our study suggests that the habitat origin of the ERM fungi may be a factor in conferring drought resistance to velvetleaf blueberry plants. However, a systematic study involving more ERM fungi species should be designed in the future to address this hypothesis.

In our study, shoot water potentials were significantly higher in well-watered lowland seedlings inoculated with *Pezicula ericae*, *Pezoloma ericae*, and *Oidiodendron maius* compared with non-inoculated plants, but there was no significant effect of fungal inoculation on shoot water potentials in the upland plants. Since there were either no significant effects of inoculation on transpirational water loss in well-water lowland plants by these fungal species, the results point to their effectiveness in water uptake and transport to the leaves under well-watered conditions as reported for other mycorrhizal associations [9,13,34].

Our results demonstrate that shoot water potentials sharply declined in plants subjected to drought stress. However, they were higher in all inoculated upland drought-stressed seedlings compared with drought-stressed non-inoculated controls. In lowland plants, *Pezoloma ericae* and *Meliniomyces variabilis* had no significant effect on shoot water potentials of drought-stressed plants. The maintenance of higher shoot water potentials in inoculated plants could be due to decreased water loss or increased root water uptake and transport through the direct contribution of fungal hyphae or indirect effects on the root water transport properties [8]. Since, compared with non-inoculated control, E was significantly higher only in drought-stressed upland plants inoculated with *Pezicula ericae* and *Pezoloma ericae*, it appears that the effect of inoculation on shoot water potentials was likely due to the effect on root hydraulics. The effect of ECM and AM associations on plant water uptake may involve an expansion of the root system [11,34], as also evidenced in our study by the increased root dry weights, especially in well-watered plants and in drought-stressed plants inoculated with *Pezicula ericae*. Both ECM and AM also enhance the aquaporin-mediated water transport in the root tissues and fungal hyphae through their effect on the aquaporin gene expression [9,13,15,35]. Water potentials can also reflect possible differences in osmotic potentials, due to osmotic adjustment in response to drought. Although mycorrhizal symbiosis can stimulate E of the host plants by improving root hydraulic conductivity to enhance water transport [34,36,37] in ECM plants, the effects on E and root hydraulic conductivity were found to vary depending on the fungal and plant species [38,39,40]. Although the ecological and functional reasons for these differences are not clear, they may likely involve the effectiveness of the fungus to enhance the aquaporin-mediated root water flow [41].

The effects of different fungal inocula on Pn in well-watered plants did not closely follow the patterns observed for E. With the exception of *Pezoloma ericae* in upland and lowland plants and *Meliniomyces variabilis* in upland plants, fungal inoculation significantly increased Pn under well-watered conditions compared with the non-inoculated control. Similarly to E, drought-stressed plants suffered from significant decreases in net photosynthetic rates (Pn). However, contrary to E, both lowland and upland drought-stressed plants inoculated with *Pezicula ericae* and *Pezoloma ericae* had significantly higher Pn compared with the non-inoculated plants. These results suggest that the effect of ERM on Pn also likely involved more than the stomatal factors. In addition to their effect on Pn through the improvement of water status, mycorrhizal fungi can affect Pn by facilitating nutrient uptake and increasing leaf chlorophyll concentrations [42,43,44]. However, in our study, fungal inoculation had only minor and not statistically significant effects on leaf chlorophyll concentrations, suggesting that other factors played a role in the observed differences in Pn between the different inoculation treatments and non-inoculated plants.

## 4. Materials and Methods

### 4.1. Plant Material

The velvetleaf blueberry (*Vaccinium myrtilloides* Michx.) seeds were collected from plants growing in wet hummocky areas in bogs (lowland plants) and dry sandy areas (upland plants) in boreal forest sites near Fort McMurray, AB, Canada. The sites did not significantly vary in their elevations. The seeds were surface-sterilized with 1% sodium hypochlorite for 2 min, followed by 70% ethanol for 5 min, and rinsed in autoclaved water. The seeds were germinated in the autoclaved (2 × 2 h) sand and commercial peat (Sun Gro Horticulture, Seba Beach, AB, Canada) mixture (2:1, by volume) in sterilized containers (72 holes Traditional 1206 Sheet Inserts, T.O. Plastics, Inc., Clearwater, MN, USA). The pH was adjusted to 4–4.5 with 20% H_2_SO_4_, and the soil moisture was maintained by adding deionized water as required. After about four months, the seeds germinated, and the germinants were transferred to individual square pots (9 × 9 × 9 cm) containing the same autoclaved growth medium as described above. Plants were grown in the controlled-environment growth room at the 18-h photoperiod, 400 of μmol m^−2^ s^−1^ photosynthetic photon flux density (PPFD), and day/night temperature of 22/18 °C. They were fertilized weekly with commercial 28-10-10 (N-P-K) Miracle-Gro acid fertilizer (The Scotts Company, LLC, Marysville, OH, USA, pH 4.5). Fertilization stopped two weeks before the fungal inoculation.

### 4.2. Isolation and Identification of ERM Fungi

ERM fungi were isolated from the roots of velvetleaf blueberry (*Vaccinium myrtilloides* Michx.) and Labrador tea [*Rhododendron groenlandicum* (Oeder) Kron & Judd] plants growing in the wet hummocky areas in bogs (lowland) and dry sandy areas (upland) in the boreal forest sites near Fort McMurray, AB, Canada. Plant seeds for the study were also collected from these two areas (Table S2).

Root samples from six plants of each species from each location (upland and lowland) were excised, washed in distilled water, cut into 5- to 7-cm-long segments, surface-sterilized with 1% sodium hypochlorite followed by 70% ethanol, and rinsed with autoclaved distilled water. To isolate the ERM fungi, 10 root segments from each plant were placed separately on plates with the autoclaved modified Melin-Norkans medium (MMN) with 1.5% agar and potato dextrose agar (PDA) medium. Fungal colonies were grown on plates and subcultured for fungal identification. Molecular identification of the ERM fungi present on the plates was carried out as described earlier [27,45] after extracting total genomic DNA using Sigma Extract-N-Amp Tissue Kit, following the manufacturer’s instructions (Sigma-Aldrich, St. Louis, MO, USA). Thus, a total of 114 DNA extracts were obtained from fungal colonies grown on plates from the root tips. The internal transcribed spacer (ITS) of the nuclear ribosomal DNA (rDNA) was amplified using the ITS1F and ITS4 primers [46,47]. All PCR products were visualized on a 1% agarose gel and purified using ExoSAP-IT (USB, Cleveland, OH, USA) and, when necessary, by gel incision following the manufacturer’s protocol (Qiagen, Toronto, ON, Canada). Sanger sequencing was carried out in one direction using the Big Dye Terminator Sequencing Mix (v. 3.1, Life Technologies Corporation, Carlsbad, CA, USA) with the same PCR forward primer at a final concentration of 0.1 μM. The resulting products were precipitated using EDTA/ethanol following the manufacturer’s instructions. The sequence was then searched in the database using the Basic Local Alignment Search Tool on the NCBI website [48].

### 4.3. Fungal Cultures

Following identification, the following isolates of the most abundant fungi were selected for the study according to the fungal colonization frequency in roots: *Pezicula ericae* (isolate # 38), *Pezoloma ericae* (isolate # 50), *Meliniomyces variabilis* (isolate #81), and *Oidiodendron maius* (isolate # 96) (Table S2). The fungi were cultured on the PDA medium solidified with 1.5% agar before being transferred to the liquid MMN medium for two weeks in the dark and at room temperature before root inoculation.

### 4.4. Root Inoculation and Drought Treatment

After two weeks of growth in the liquid medium, the fungal hyphae were filtered and washed with autoclaved deionized water. They were then homogenized in a blender and suspended in autoclaved water to the mycelial concentration of 40 (±5) g L^−1^. Fungal inoculum was added to the soil with two-month-old seedlings that were 2-3-cm tall at the time of inoculation. Four holes were made in the soil around the roots and 5 to 7 mL of liquid inoculum was injected into each hole with a pipette. The non-inoculated seedlings were provided with the same amount of autoclaved deionized water to serve as non-inoculated control. Following inoculation, the plants were grown for 3 months. They were watered every 3 days and fertilized weekly with commercial Miracle-Gro acid fertilizer before the start of the drought treatment.

There were 36 upland and 36 lowland velvetleaf blueberry plants per inoculation (four ERM fungi and non-inoculated control) for the total of 360 plants. Three months after the inoculation, the plants were further divided into two equal groups. The first group of 18 plants from each population and each inoculation treatment was subjected to three cycles of drought treatment, and the second group of 18 plants was watered every second day to serve as drought control. In each cycle, watering was withheld for 7 days and followed by re-watering to runoff, for the total of 21 days of the drought treatment.

### 4.5. Volumetric Soil Water Content

Volumetric soil water content was measured with the 1502C Metallic Time-Domain Reflectometer (TDR) instrument (Tektronix, Inc., Beaverton, OR, USA). A three-rod (1.5 mm in diameter, 10-cm long) TDR stainless steel probe was vertically inserted into the soil of 6 pots per treatment [49]. The measurements were carried out daily, starting on day 4 of the first drought cycle, between 9:30 and 11:30 a.m. The volumetric soil water content was determined based on the below equation calibrated for the water volume and soil volume ratio [49].

Volumetric soil water content (V/V) × 100% = 3.5922 × (d_2_ − d_1_) − 0.1727 (1)

where d_1_ is the first peak generated by the voltage change in the wave signal due to the connection between the coaxial cable, and the rod of the probe and d_2_ is the second peak generated when the wave signal reaches the end of the rods in the soil encountering an open circuit.

### 4.6. Root Colonization by ERM Fungi

Hair roots from 6 plants (10 roots per plant) per treatment (*n* = 6) were excised at the end of the drought treatment and fixed in FAA (formaldehyde:ethanol:acetic acid:water, 10%:50%:5%:35%). Roots were rinsed twice in distilled water to remove the FAA solution, clarified with 10% KOH at 60 °C for 1 h, and washed twice with distilled water followed by 5% acetic acid. The cleared roots were stained with black ink (Sheaffer, Shelton, CT, USA) in 5% acetic acid at 60 °C for 20 min and rinsed with distilled water. The roots were cut into approximately 1-cm-long segments, and 10 randomly selected segments from each seedling were mounted on microscope slides to examine for root colonization by counting the number of coils. Root colonization by the ERM fungi was examined with the light microscope according to Trouvelot et al. (1986) [50]. The root samples were rated from 0 to 5 according to the ERM intensity in the root samples [50]. Colonization intensity (M%) was calculated for all treatments using the following equation (Trouvelot et al., 1986 [50]:

M (%) = (95n5 + 70n4 + 30n3 + 5n2 + n1)/(ns total) (2)

where n1–5 is the number of root segments rated 1–5 and ns total is the total number of root segments

### 4.7. Plant Mortality and Dry Weights

Plant mortality in each inoculation group was determined at the end of the experiment. For growth determination, plants were harvested at the end of the drought treatments, and their roots, leaves, and stems separated and weighed to determine fresh weights (*n* = 6). For dry weights, the stems and roots were oven-dried at 70 °C, and the leaves were freeze-dried for 72 h and weighed. The weights of leaves and stems from each plant were combined to calculate shoot dry weights.

### 4.8. Gas Exchange

Net photosynthetic (Pn) and transpiration (E) rates were measured at the end of the drought treatment in six plants per treatment combination, and their weights added to the remaining leaves for fresh and dry weight determinations (*n* = 6) using the LI-6400XT portable open-flow photosynthesis system equipped with a red/blue LED light source (LI-COR, Inc., Lincoln, NE, USA). The PPFD was set at 400 μmol∙m^−2^∙s^−1^, leaf temperature at 28 °C, and the reference CO_2_ concentration was maintained at 400 μmol∙mol^−1^ using the 6400-01 CO_2_ mixer. All measurements were carried out between 4 and 8 h after the onset of the photoperiod on the upper fully expanded leaves. Leaf areas were calculated following computer scanning using the Sigmascan Pro 5.0 computer software (Systat Software, San Jose, CA, USA).

### 4.9. Leaf Chlorophyll Concentrations

Leaf chlorophyll concentrations were determined in the upper fully expanded leaves harvested from six randomly selected seedlings per treatment (*n* = 6). Leaves were freeze-dried and ground in a Thomas Wiley Mini-Mill (Thomas Scientific, Swedesboro, NJ, USA). Chlorophyll was extracted from pulverized leaf samples (10 mg dry weight) with 8 mL of dimethyl sulfoxide (DMSO) at 65 °C for 22 h. Chlorophyll concentrations were measured in DMSO extracts with a spectrophotometer (Ultrospec, Pharmacia LKB, Uppsala, Sweden), at 648 nm and 665 nm for chlorophyll-a and chlorophyll-b, respectively. Total chlorophyll concentrations were calculated using the Arnon’s equation [51].

### 4.10. Shoot Water Potentials

Midday shoot water potentials (ψ_w_) were measured in six plants per treatment (*n* = 6) that were also used for the measurements of chlorophyll concentrations and root colonization) using a Scholander-type pressure chamber (PMS Instruments, Corvallis, OR, USA). The shoots were excised and immediately placed in the pressure chamber for the measurements [14].

### 4.11. Data Analysis

The data for shoot, root, and total dry weights, shoot/root ratio, Pn, E, shoot water potential, and chlorophyll concentrations in each velvetleaf blueberry populations were analyzed by one-way ANOVA. The three-way ANOVA was used for the comparison of treatments, including drought, inoculation, and plant population, using the IBM SPSS statistical package (version 21, IBM Corporation, Armonk, NY, USA). Separation of means was conducted based on Duncan’s multiple range test at the 0.05 significance level. Data were log_10_ transformed when they did not meet the normality and homoscedastic postulates.

## 5. Conclusions

In summary, our results demonstrated that non-inoculated upland velvetleaf blueberry plants had higher shoot, root, and total dry weights under well-watered conditions compared with the lowland plants. However, these differences were offset by the inoculation of plants with ERM fungi, some of which also significantly improved drought resistance characteristics of upland and lowland plants, supporting our original hypothesis. The growth of lowland plants, as evidenced by plant dry weights, benefitted relatively more from the ERM inoculation compared with the upland plants under well-watered conditions, but this was no longer apparent in plants subjected to the drought treatment. Also, there were no major differences in the effects of different ERM fungal species on drought responses of upland and lowland plants. *Pezicula ericae* appeared to be more beneficial compared with the other examined ERM fungi to most of the studied growth and physiological parameters in both upland and lowland velvetleaf blueberry seedlings and under both well-watered and drought stress conditions. The ERM inoculation of lowland plants increased shoot water potential under well-watered conditions, and the inoculation with *Pezicula ericae* was especially effective in maintaining higher shoot water potentials, Pn, and E in plants subjected to drought stress. Further studies are required to determine the importance of the fungal habitat origin in conferring drought resistance to plants.

## Figures and Tables

**Table 1:** Root colonization intensity (M) in non-inoculated upland and lowland velvetleaf blueberry plants and in plants inoculated with different species of ERM fungi that were either well-watered or subjected to three cycles of drought treatment for 21 days. Means (*n* = 6) ± SE are shown. Different letters indicate statistically significant differences between plants inoculated with different ERM fungi and non-inoculated control determined by the three-way ANOVA (Duncan’s test *p* ≤ 0.05).

Inoculation	Treatment	Population	M% ± SE	
Non-inoculated	Drought	Upland	20.7 ± 6.6	a
		Lowland	17.4 ± 3.8	
	Well-watered	Upland	17.5 ± 3.8	
		Lowland	14.7 ± 4.9	
*Pezicula ericae*	Drought	Upland	55.8 ± 3.2	c
		Lowland	63.8 ± 5.0	
	Well-watered	Upland	69.3 ± 4.5	
		Lowland	71.1 ± 4.4	
*Pezoloma ericae*	Drought	Upland	67.3 ± 4.0	b
		Lowland	51.3 ± 4.2	
	Well-watered	Upland	56.9 ± 3.8	
		Lowland	53.3 ± 7.0	
*Meliniomyces variabilis*	Drought	Upland	55.2 ± 4.1	bc
		Lowland	51.8 ± 3.4	
	Well-watered	Upland	62.5 ± 6.3	
		Lowland	78.2 ± 4.2	
*Oidiodendron maius*	Drought	Upland	66.0 ± 4.1	d
		Lowland	78.7 ± 3.8	
	Well-watered	Upland	77.4 ± 2.7	
		Lowland	78.3 ± 3.3	

**Table 2:** Mortality (%) of non-inoculated (NI) upland (UB) and lowland (LB) velvetleaf blueberry and plants inoculated with different ERM fungi and subjected to three cycles of drought treatment for 21 days (*n* = 18).

Plant Group	NI	*Pezicula ericae*	*Pezoloma ericae*	*Meliniomyces variabilis*	*Oidiodendron maius*
LB	33.3	5.6	5.6	16.7	11.1
UB	22.2	11.1	11.1	5.6	11.1

## Data Availability

The data are available upon request.

## Supplementary Materials

The following are available online at https://www.mdpi.com/article/10.3390/plants10122786/s1, Table S1. Three-way ANOVA analysis results of watering treatment (drought and well-watered), plant provenance (upland and lowland), and fungal inoculation) effects on the measured parameters, Table S2. Taxonomic affinities of the four ERM fungal isolates that were selected for the study as inferred from BLAST queries of ITS sequences in GenBank.
