# Supplementary material for: Inoculation with Ericoid Mycorrhizal Associations Alleviates Drought Stress in Lowland and Upland Velvetleaf Blueberry (Vaccinium myrtilloides) Seedlings
Source: Plants (Basel). 2021 Dec 16;10(12):2786. doi: 10.3390/plants10122786 (PMC8704242; doi:10.3390/plants10122786)
Supplement: Supplementary file 1 [file plants-10-02786-s001.zip › plants-1458617-supplementary.pdf]

Table S1. Three-way ANOVA analysis results of watering treatment (drought and well-watered), plant provenance (upland and lowland), and fungal inoculation effects on the measured parameters.

Source	Significance Value										
	Shoot Dry Weights	Root Dry Weights	Total Dry Weights	S/R Ratios	Tissue Water Content	Leaf Areas	Water Potentials	Leaf Chlorophyll Concentrations	Pn	E	M
Drought	<0.0001	<0.0001	<0.0001	0.012	<0.0001	<0.0001	<0.0001	0.088	<0.0001	<0.0001	0.007
Population	0.143	0.605	0.208	0.003	0.272	0.19	0.072	0.728	0.724	0.393	0.79
Fungi	<0.0001 [a]	<0.0001 [b]	<0.0001 [c]	0.114	0.316	<0.0001 [d]	<0.0001 [e]	0.012 [f]	<0.0001 [g]	0.057	<0.001
Drought x Population	0.276	0.275	0.264	0.803	0.104	0.7	<0.0001	0.051	0.065	0.611	0.404
Drought x Fungi	0.056	0.026	0.053	0.076	0.162	0.117	<0.0001	0.931	0.15	0.295	0.01
Population x Fungi	0.735	0.341	0.683	0.527	0.568	0.227	0.074	0.963	0.225	0.051	0.048
Drought x Population x Fungi	0.712	0.636	0.703	0.504	0.015	0.735	0.063	0.259	0.573	0.495	0.128

Note: Different letters indicate significant differences between different ERM fungi.

Table S2. Taxonomic affinities of the four ERM fungal isolates that were selected for the study as inferred from BLAST queries of ITS sequences in GenBank.

Sample ID	BLAST Result					Plant Origin
	Closest Match in GenBank (accession no.)	ITS-PCR Products (bp)	Query Coverage	Identity	E Value	
#38	*Pezicula ericae* (NR_155653)	547	99%	99%	0	upland velvetleaf blueberry
#50	*Pezoloma ericae* (KY315940)	538	100%	99%	0	lowland velvetleaf blueberry
#81	*Meliniomyces variabilis* (HM190126)	537	100%	99%	0	upland Labrador tea
#96	*Oidiodendron maius* (MH860824)	537	99%	99%	0	lowland Labrador tea
